# Unique Temporal Expression of Triplicated Long-Wavelength Opsins in Developing Butterfly Eyes

**DOI:** 10.3389/fncir.2017.00096

**Published:** 2017-11-29

**Authors:** Kentaro Arikawa, Tomoyuki Iwanaga, Motohiro Wakakuwa, Michiyo Kinoshita

**Affiliations:** ^1^Laboratory of Neuroethology, Department of Evolutionary Studies of Biosystems, Graduate University for Advanced Studies (SOKENDAI), Hayama, Japan; ^2^Graduate School of Integrated Science, Yokohama City University, Yokohama, Japan

**Keywords:** insect, compound eye, ommatidium, photoreceptor, development, eye disk, visual pigment, rhodopsin

## Abstract

Following gene duplication events, the expression patterns of the resulting gene copies can often diverge both spatially and temporally. Here we report on gene duplicates that are expressed in distinct but overlapping patterns, and which exhibit temporally divergent expression. Butterflies have sophisticated color vision and spectrally complex eyes, typically with three types of heterogeneous ommatidia. The eyes of the butterfly *Papilio xuthus* express two green- and one red-absorbing visual pigment, which came about via gene duplication events, in addition to one ultraviolet (UV)- and one blue-absorbing visual pigment. We localized mRNAs encoding opsins of these visual pigments in developing eye disks throughout the pupal stage. The mRNAs of the UV and blue opsin are expressed early in pupal development (pd), specifying the type of the ommatidium in which they appear. Red sensitive photoreceptors first express a green opsin mRNA, which is replaced later by the red opsin mRNA. Broadband photoreceptors (that coexpress the green and red opsins) first express the green opsin mRNA, later change to red opsin mRNA and finally re-express the green opsin mRNA in addition to the red mRNA. Such a unique temporal and spatial expression pattern of opsin mRNAs may reflect the evolution of visual pigments and provide clues toward understanding how the spectrally complex eyes of butterflies evolved.

## Introduction

The structure and function of visual systems vary depending on animals’ habitats and foraging strategies. For example, flower-visiting diurnal insects often have spectrally-richer eyes than nocturnal non-flower visitors whose eyes have higher absolute sensitivity instead. What is the proximate cause of creating such evolutionary diversity? How did some eyes become spectrally complex compared to their simpler ancestors?

**Figure 1:**
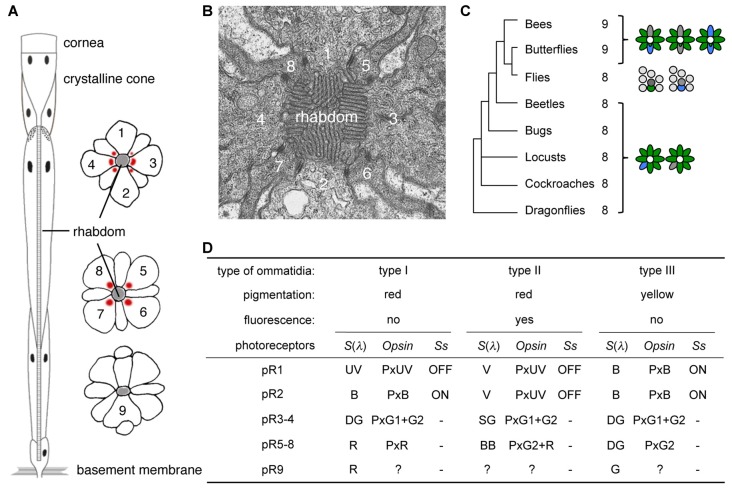
Butterfly ommatidia. **(A)** Diagram of a tiered ommatidium. Nine photoreceptors (1–9) form a phototransductive rhabdom along the central axis. **(B)** Transverse section of distal rhabdom of adult. **(C)** Insect phylogeny and photoreceptor number per ommatidium. Right diagrams show typical ommatidial types. **(D)** Three ommatidial types of *Papilio xuthus*. Information about pR9 photoreceptors is limited, but see also Figure 5 in Briscoe (2008) for opsins expressed in the pR9 cells of *Papilio glaucus*. *Ss*, Spineless; *S*(*λ*), spectral sensitivity.

Compound eyes consist of thousands of ommatidia each housing several photoreceptor cells, with the precise number of photoreceptors differing among species (Figure 1; Friedrich et al., 2011; Wernet et al., 2015). The ommatidia can typically be divided into two or three spectrally distinct types according to the spectral sensitivities of their photoreceptors. Interestingly, species with two ommatidial types in their main retina, such as locusts (Schmeling et al., 2014), leafhoppers (Wakakuwa et al., 2014), and flies (Franceschini et al., 1981; Chou et al., 1999) have eight photoreceptors (R1-8) in each ommatidium. On the other hand, the ommatidia of butterflies and bees typically bear nine photoreceptor cells (R1-9), and their main retinas contain three types of ommatidia (Figures 1A,C).

The ninth photoreceptor in butterflies and bees serves to enhance their eyes’ spectral sophistication. During development, the eyes of butterflies contain two photoreceptor precursors per ommatidium that express the transcription factor Prospero, which is expressed in the precursor of the R7 photoreceptor in *Drosophila melanogaster* (Cook et al., 2003). Once expressing Prospero, a subset of *Drosophila* R7 photoreceptors (dR7s) then stochastically expresses the transcription factor Spineless while others do not, and this binary distinction divides the ommatidia into types (Wernet et al., 2006). Because butterfly ommatidia contain two dR7-like Prospero-positive cells, there are three possible combinations: ON-OFF, OFF-OFF and ON-ON, and these Spineless expression patterns indeed correspond precisely with the three types of ommatidia in the Japanese yellow swallowtail butterfly, *Papilio xuthus* (Figure 1D; Arikawa, 2003; Perry et al., 2016).

Spectral richness of compound eyes is often achieved via visual pigment gene duplication (Briscoe, 2008), which facilitates improved color vision. The ancestral insect eye likely expressed three visual pigment opsins, corresponding to visual pigments absorbing short-wavelength or ultraviolet (UV), middle-wavelength or blue (B), and long-wavelength or green (G; Chang et al., 1996; Townson et al., 1998; Wakakuwa et al., 2005). This presumed ancestral scheme still exists in honeybees, with these visual pigments forming the physiological basis of their UV-B-G trichromacy (von Helversen, 1972). However, butterflies often have more than three opsins: for example, *Papilio xuthus* has two G opsins (G1 and G2) and one red (R) opsin in addition to the UV and B opsins (Kitamoto et al., 1998), due to repeated gene duplication events (Briscoe, 1999). As a result, the eyes of *Papilio xuthus* are more spectrally complex, containing six classes of spectral receptor. The R opsin is expressed in a subset of photoreceptors, which are thus red sensitive (Figure 1). Even with six classes of spectral receptors in the eye, color vision is not necessarily hexachromatic: the wavelength discrimination function indicates that *Papilio* color vision is UV-B-G-R tetrachromatic (Koshitaka et al., 2008).

Some of the new opsins acquired via gene duplication processes are coexpressed with other opsins within single photoreceptors, enhancing the photoreceptors’ spectral variation further. Coexpression of opsins contradicts the classical one cell-one opsin concept, but accumulating evidence indicates that this phenomenon is much more common than had previously been thought, both in invertebrates and vertebrates. These opsin-coexpressing photoreceptors often exist in retinal margins or around the border of distinct retinal regions, suggesting that they may be merely imperfectly differentiated (Röhlich et al., 1994; Makino and Dodd, 1996; Parry and Bowmaker, 2002; Hu et al., 2011). However, recent studies have shown that such photoreceptors occupy specific position in the retinal mosaic, implying functional importance (Mazzoni et al., 2008; Rajkumar et al., 2010; Dalton et al., 2014; Chen et al., 2016; McCulloch et al., 2017). For example, the *Papilio* R5-8 (pR5-8) photoreceptors of type II ommatidia (Figure 1D) are broad-band receptors, coexpressing G2 and R opsins (Arikawa et al., 2003). The green sensitive pR3 and pR4 photoreceptors coexpress G1 and G2 across all but the limited dorsal region of the eye (Kitamoto et al., 1998).

Presently, it is poorly understood how the coexpression of multiple opsins is controlled after ommatidial fate determination, and how such complex eye organization has evolved in butterflies. To investigate these issues, we studied the anatomy of developing compound eyes in *Papilio xuthus* with particular attention to the expression of opsin mRNAs in photoreceptor precursors.

## Materials and Methods

### Animals

We used Japanese yellow swallowtails, *Papilio xuthus*, from a stock culture derived from individuals captured in Kanagawa, Japan. Larvae were reared on fresh citrus leaves under a light regime of 14 h light:10 h dark at 25 ± 1°C, which produces non-diapausing pupae. The pupae were kept under the same conditions. Adult females emerge on the 11th day after pupation; the pupal period is 10 days. The pupal period of males is 1 day shorter, and we used only females in this study for simplicity and clarity. We used at least five individuals per each individual stage.

### Anatomy

To determine the stages of eye development, we studied the morphology of eye disks (pupal eye tissue) under a dissecting microscope at 24 h intervals starting from the day of pupation (1 day pupa) to 1 day before adult eclosion (10 day pupa).

For light and electron microscopy, eye disks were prefixed in 2% glutaraldehyde, 2% paraformaldehyde in 0.1M sodium cacodylate buffer (CB, pH 7.4) for 2 h at room temperature and postfixed in 2% osmium tetroxide in CB for 2 h at room temperature. The tissues were then dehydrated with an acetone series and embedded in Epon. Sections of 5 μm thickness were stained with Azur-II for light microscopy. Ultrathin sections were stained with uranyl acetate and were observed in Hitachi H-7650 electron microscope. For immunohistochemistry, eye disks were fixed in 4% paraformaldehyde in 0.1M sodium phosphate butter (pH 7.4) for 2–6 h on ice; see Perry et al. (2016) for the labeling procedure.

### RT-PCR Analysis

The *Papilio* retina contains five opsin mRNAs each encoding *Papilio xuthus* ultraviolet-absorbing (PxUV), PxB (blue), PxL1 (green), PxL2 (green) and PxL3 (red) (Kitamoto et al., 1998, 2000). We determined the post-pupation day at which each of these opsin mRNAs became detectable by reverse transcription polymerase chain reaction (RT-PCR). Total RNA was extracted from the eye tissues of the pupa every 24 h using the RNAgents Total RNA Isolation Kit (Promega), and cDNA was synthesized using oligo-dT primer by reverse transcription. The primers were designed to amplify 200–300 bp fragment of one of the opsin mRNA. The sequences of the primers are as follows: PxUVF (PxUV-Forward), TGAAT TCACA TATAC TGACC CAACG CG; PxUVR (PxUV-Reverse) GGAAA GCTTT CCATT ATTCA CGCCA GTTC; PxBF, AGAAT TCTCC AACGA ACGAT GCAAT CG; PxBR, CGAAA GCTTT CGGAG TCCAT ACAAC AAGC; PxL1F, TGAAT TCAAC GACGA CGAAT GTTTG CG; PxL1R, TAAAA GCTTT TACTA TCGCA GGCTA AC; PxL2F, GGAAT TCCCC TAAGG ATCTG ATACT GC; PxL2R, ACCAA GCTTG GTACA CAGCT TGTTT CATC; PxL3F, TGAAT TCAAC CAACG ATGAC GACTT GG; PxL3R, GATAA GCTTA TCACA CGAGG ATAGT AGGG. We also used primer sets for amplifying cDNA of actin (forward = CAYAC NGTIC CNATH TAYGA RGG; reverse = TCIGC DATNC CNGGR TACAT NGT).

### *In Situ* Hybridization

The pupal eye tissues were fixed in 4% paraformaldehyde in 0.1 M buffered sodium phosphate (pH 7.2, PB) for 0.5–2 h at 25°C. After dehydration with an ethanol series, they were embedded in paraplast, sectioned at 8 μm thickness, mounted on poly-L-lysine-coated slide and dried overnight at 37°C.

Probes for *in situ* hybridization were designed to hybridize to about 400 bases of the mRNA in the non-coding region downstream of the C-terminal. The corresponding cDNA region was first subcloned into pGEM-3zf(+) vector, and then digoxigenin (DIG)-labeled cRNA was generated using the DIG-RNA labeling kit (Roche).

The sections were deparafinized and treated with hybridization solution (300 mM NaCl, 2.5 mM EDTA, 200 mM Tris–HCl (pH 8.0), 50% formamide, 10% dextran sulfate, 1 mg/ml yeast tRNA, 1× Denhardt’s medium), containing 0.5 mg/ml of the cRNA probe at 45°C overnight. After a brief rinse, the sections were incubated in 50% formamide in 2× SSC (saline sodium citrate buffer) at 55°C for 2 h, and then treated with RNase (10 mg/ml) at 37°C for 1 h. The probes were further visualized by anti-DIG immunocytochemistry.

## Results

### Cellular Organization

**Figure 2:**
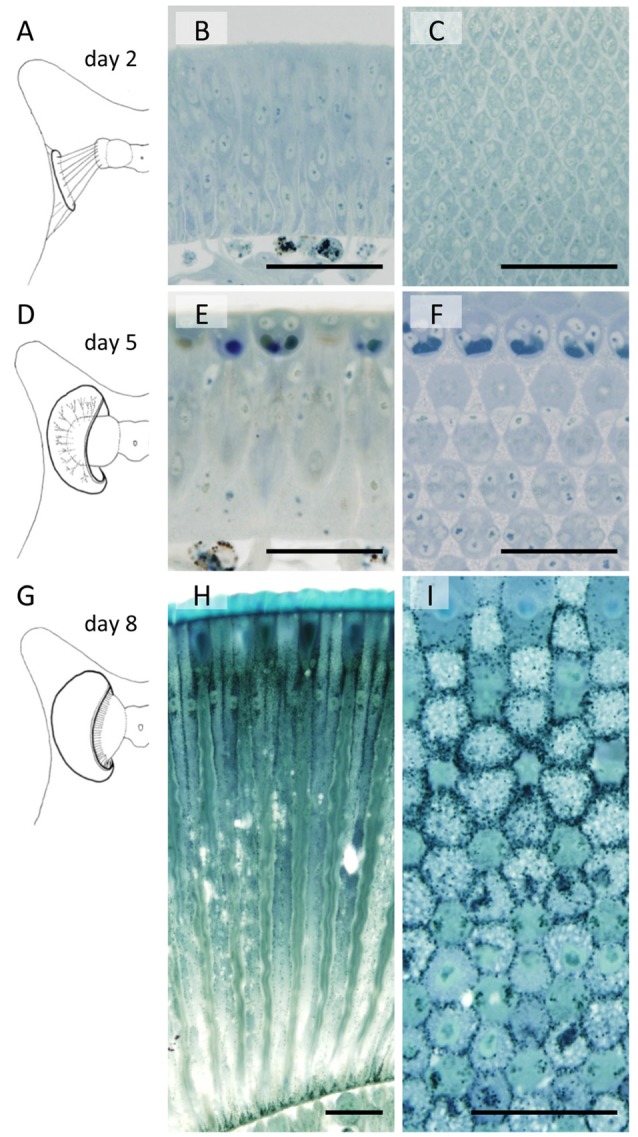
Developing compound eyes of *Papilio xuthus* in day 2 **(A–C)**, day 5 **(D–F)** and day 8 **(G–I)** pupae. General appearance of the eye disk in the pupa **(A,D,G)**, and the longitudinal **(B,E,H)** and transverse **(C,F,I)** sections. Scales = 50 μm.

The developing compound eye (eye disk) in the day 1 pupa is a sheet of tissue attached to the pupal cuticle by connective tissue (Figure 2A). It expands and detaches from the cuticle by day 4. The characteristic dome shape of the mature eye is formed in day 5 (Figure 2D) and pigmentation starts on day 8 (Figures 2H,I).

Figure 2 also shows sections of developing eye tissues, cut parallel (middle column) and perpendicular (right column) to the ommatidial optical axis. Because of the curvature of the tissue, the perpendicular sections are actually slightly oblique, showing transverse views of ommatidia at different levels within single sections.

By day 2 we can already identify hexagonally arranged cell groups, corresponding to single ommatidia (Figures 2A–C). However, cell types (e.g., photoreceptors or pigment cells) are not clear from the conventional histology at the light microscopic level. On day 5, precursors of crystalline cone cells, photoreceptor cells, and pigment cells can begin to be distinguished according to the positions of the nuclei (Figures 2E,F). The basic compartments of the ommatidia are all clearly identifiable by day 6. The length of the ommatidia are about 130 μm, which increases over the next 3 days, reaching more than 450 μm on day 8 with a characteristic two-tiered configuration (Figures 1A, 2G–I). This developmental process is similar to that of *Manduca sexta* (Monsma and Booker, 1996).

### Ultrastructure

**Figure 3:**
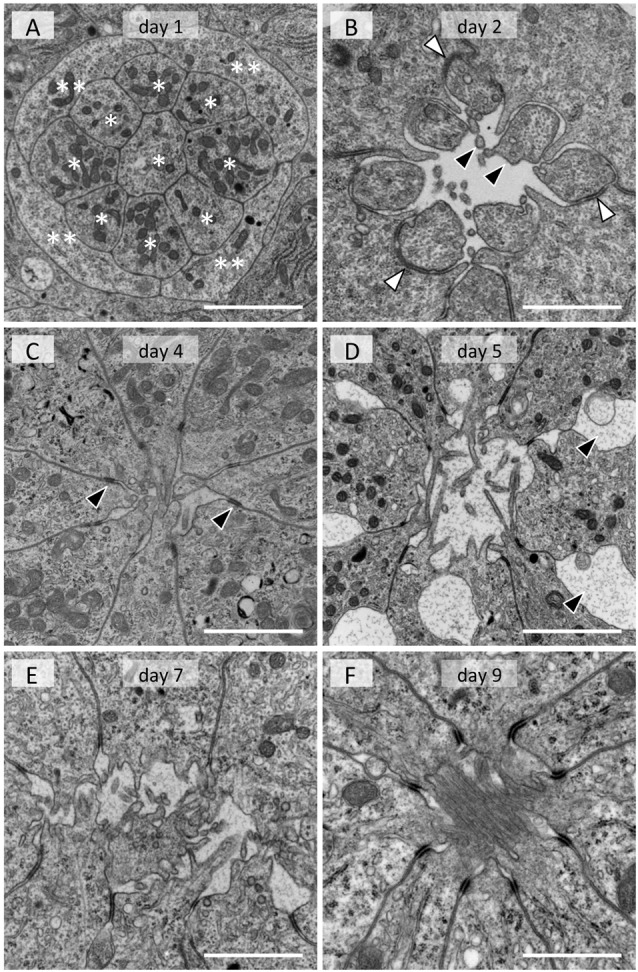
Electron micrographs of developing rhabdom in the eye disk from pupae. **(A)** Day 1. Eight columnar cells (*) and flat cells (**) are evident. **(B)** Day 2. Membrane of photoreceptor precursors start evaginating (black arrowheads). White arrowheads indicate desmosome-like junctions. **(C)** Day 4. Arrowheads indicate interphotoreceptor junctions as in adult ommatidia (see Figure 1B). **(D)** Day 5. Interphotoreceptor spaces enlarge (arrowheads). **(E)** Day 7. **(F)** Day 9. Scales = 2 μm.

In the adult, photoreceptor cells extend numerous tightly packed microvilli towards the center of the ommatidium, forming a fused rhabdom about 2 μm in diameter (Figure 1B). In the day 1 tissue, groups of 13 cells are evident at the electron microscopic level. A group always has a columnar cell in the center, which is surrounded by eight other columnar cells (single asterisks in Figure 3A). Four cells that appear flat in transverse sections wrap around the group of nine columnar cells (double asterisks in Figure 3A). The space between the cell groups is filled with rough ER-rich cells. Presumably, the columnar, flat and rER-rich cells respectively correspond to photoreceptor, cone and pigment cell precursors. Therefore we hereafter refer to these precursor cells as photoreceptors, cone cells and pigment cells for simplicity.

On day 2, a cavity appears in the center, surrounded by eight photoreceptor cells (Figure 3B). The cell which was in the center on the previous day (most likely the precursor of the basal photoreceptor R9) moves to the proximal end of the extending ommatidium. The eight remaining photoreceptors start to evaginate irregular processes towards the cavity (black arrowheads in Figure 3B). The cone cells enlarge and interpose between photoreceptor cells, forming belt desmosome-like junctions with the photoreceptor cells in places (white arrowheads in Figure 3B).

The cross sectional area of the photoreceptor cells increases on day 4 (Figure 3C). The cone cell cytoplasm between the photoreceptors disappears and thus the photoreceptor cells come into direct contact, forming belt desmosomes between them as in the adult (arrowheads in Figure 3C). The photoreceptor cells continue to extend processes into the cavity.

The most notable feature of the day 5 tissues is the enlarged inter-photoreceptor spaces (arrowheads in Figure 3D), indicating that the packing of the photoreceptors becomes somewhat loose. The ommatidia extensively elongate from day 6 to 9, so these two phenomena may be related.

The diameter of the photoreceptor processes in the central cavity is variable on day 7 (Figure 3E), but it becomes about 80 nm, which is equivalent to the adult microvilli, by day 8. Although the cavity is mostly filled with microvilli by day 9 (Figure 3F), the arrangement of microvilli is still incomplete (see Figure 1B). The rhabdom formation is probably completed shortly after adult eclosion.

### Expression and Localization of Opsin mRNAs

**Figure 4:**
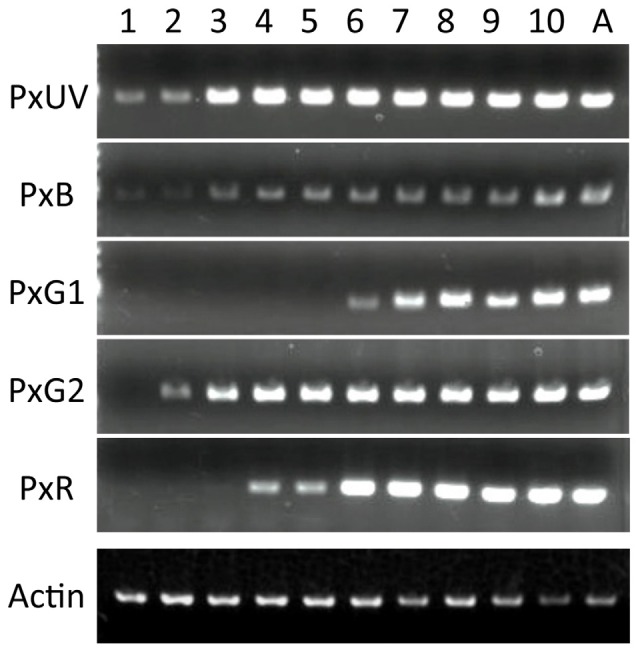
Expression analysis of five visual pigment opsins by RT-PCR. Numbers on the top indicate days after pupation, and A for adult. As a control, the actin cDNA is amplified.

Figure 4 shows the results of RT-PCR of five opsin mRNAs, as well as the mRNAs of *Papilio* actin. The concentration of the template cDNA is adjusted to 5 μg/μl, which gives stable amplification results in the actin control (see bottom row). Not all opsin mRNAs appear simultaneously. The mRNAs of PxUV and PxB are already detectable on day 1. Those of PxG2 and PxR become detectable on day 2 and day 4, respectively, but the PxG1 mRNA does not appear until day 6.

**Figure 5:**
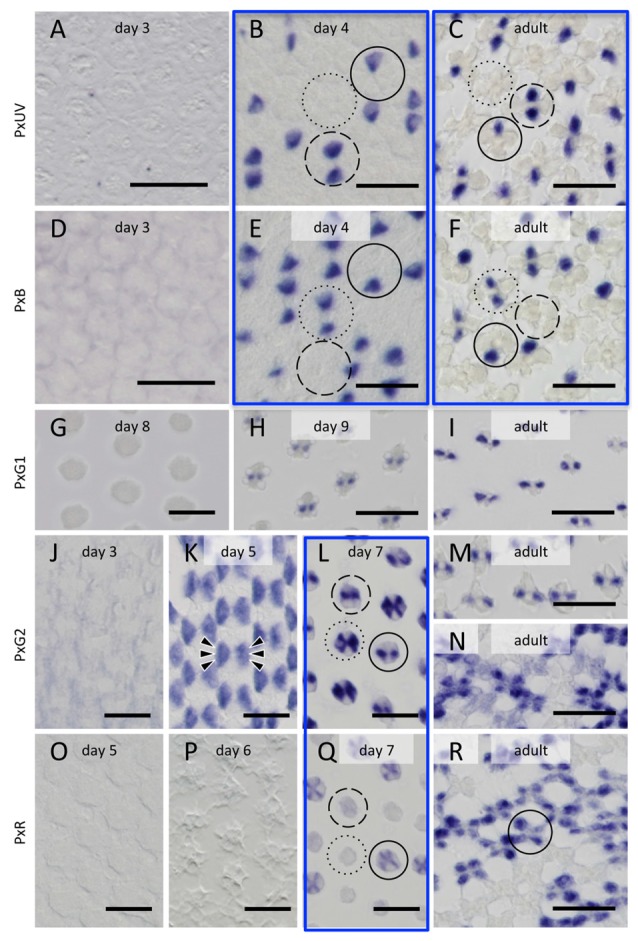
Histological *in situ* hybridization of five opsin mRNAs in the developing and adult compound eyes of *Papilio xuthus*. **(A–C)** PxUV. **(D–F)** PxB. **(G–I)** PxG1. **(J–N)** PxG2. **(O–R)** PxR. Developmental stages are indicated in each pictures. Three framed pairs are from adjacent sections labeled with different probes. Solid, broken and dotted circles indicate type I, II and III ommatidia. Arrowheads in **(K)** indicate six cells labeled. Scales = 20 μm.

We carried out histological *in situ* hybridization of five opsin mRNAs in the developing eye tissue isolated from pupae of various stages (Figure 5), and could identify signals by *in situ* hybridization 4 days after the detection of all opsin mRNAs by RT-PCR. This presumably reflects the difference in sensitivity between these two methods.

The right-most column of Figure 5 shows the distribution of opsin mRNAs in the adult, which is summarized in Figure 1D (Arikawa, 2003). The three framed pairs (b/e, c/f, l/q) are adjacent sections labeled with different probes. Solid, broken and dotted circles in these pictures indicate type I, II and III ommatidia, respectively.

On day 3, no signals are detectable. The probes specific to PxUV and PxB mRNAs give adult-like labeling on day 4 (Figures 5B,C,E,F). Three types of ommatidia are evident according to the labeling pattern in *Papilio* R1 (pR1) and pR2: PxUV-PxB (I in Figure 5B), PxUV-PxUV (II), and PxB-PxB (III). This indicates that the fates of the pR1 and pR2 photoreceptors are already determined by day 4.

The three mRNAs encoding long-wavelength opsins appear later. The expression of the PxG1 mRNA is detected only after day 9 in the pR3 and pR4 photoreceptors of all ommatidia (Figures 5G,H), as in the adult (Figure 5I). As seen in Figure 5L, these photoreceptors coexpress PxG2 mRNA (Kitamoto et al., 1998). PxG2 mRNA is first detected on day 5 in the pR3,4 distal photoreceptors and pR5-8 proximal photoreceptors of all ommatidia (6 arrowheads in Figure 5K). Because the ommatidia are still short and the tiered-configuration is not yet formed on day 5, all six of these photoreceptors are visible in a single section (Figure 5K). Intriguingly, the PxG2 labeling in pR5-8 proximal receptors disappears on day 7 in a number of ommatidia, while the labeling in pR3 and pR4 remains (solid and broken circles in Figure 5L). These pR5-8 photoreceptors in some ommatidia instead express the PxR mRNA (solid circle in Figure 5Q); these correspond to type I ommatidia in the adult. In some other ommatidia, the cells were labeled faintly with the PxR probe (broken circle in Figure 5Q); these are type II ommatidia (Figure 5N), whose pR5-8 photoreceptors are the broad-band receptors coexpressing PxG2 and PxR visual pigments (Arikawa et al., 2005).

## Discussion

### Comparison with *Drosophila* Eye Development

Development of insect compound eyes has been extensively studied in *Drosophila melanogaster* (Ready et al., 1976; Cagan and Ready, 1989). In *Drosophila*, the pupal stage lasts for 160 h at 20°C (Cagan and Ready, 1989) and the fate of ommatidia is determined early in the pupal stage (Michael Perry, personal communication; Wernet et al., 2006). The first mRNA encoding a visual pigment opsin, Rh1, then appears at 78% of the way through pupal development (pd, 78%), which is followed by the expression of mRNAs encoding Rh3, 4 and 5 at 80% pd, and Rh6 at 82% pd (Earl and Britt, 2006). Expression of the respective opsin proteins follows the same course with a slight (1%–2% pd) delay (Earl and Britt, 2006).

**Figure 6:**
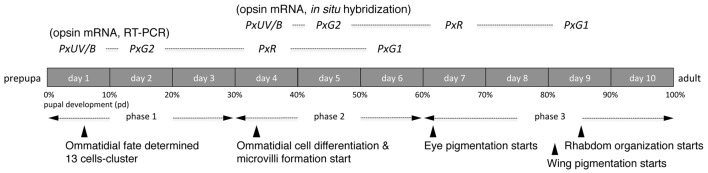
Development of the *Papilio* pupal eye disk.

Figure 6 summarizes the process of pupal development of *Papilio* in terms of the expression of opsin mRNAs. Ommatidial fate determination is completed in phase 1 as in *Drosophila* (Perry et al., 2016). Interestingly, in *Papilio* opsin mRNAs are already detectable at 10% pd via RT-PCR. It requires four more days before mRNA becomes detectable in histological *in situ* hybridization, presumably because RT-PCR is a more sensitive technique. We tried to localize opsin proteins by immunohistochemistry using specific antibodies against PxUV, PxB and PxG2 in the eye disks taken from pupae of ~60% pd. Although those antibodies successfully label subsets of photoreceptors in adults (Perry et al., 2016), we could not detect any signs of labeling in the pupal eye disks. This strongly indicates that opsin proteins are not synchronously expressed with their mRNAs, unlike in *Drosophila* (Kumar and Ready, 1995).

Rhabdomere formation happens much earlier in *Drosophila* than in *Papilio*. *Drosophila* photoreceptors already exhibit short but organized arrays of processes on the apical side by 55% pd. The developing rhabdomeral structures start to separate at 73% pd, eventually forming the open rhabdomere configuration by 90% pd (Kumar and Ready, 1995). On the other hand, the rhabdomeral microvilli of *Papilio* photoreceptors are quite immature even at 70% pd. Formation of a clear border between the rhabdomere and the photoreceptor cell body, i.e., the structure of the microvillar base, starts very late at around 90% pd (Figure 3). Opsins are essential for making the microvillar base structure in *Drosophila* (Kumar and Ready, 1995). Assuming a similar structural function of *Papilio* opsins in developing photoreceptors, the late translation of mRNAs into opsin proteins is likely related to this phenomenon.

### Temporal Coexpression of Opsin mRNAs

**Figure 7:**
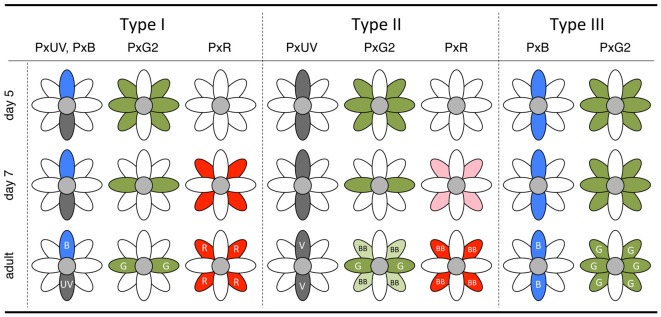
Summary diagram of the *in situ* hybridization results of day 5 and day 7 pupae with the final state in the adult.

Results from *in situ* hybridization experiments are summarized in Figure 7. The *Papilio* R9 (pR9) photoreceptor has been specified by this point (see Figure 3A), as shown via immunohistochemistry using whole-mount preparations (Perry et al., 2016). However, we could not identify these cells in the sections cut for *in situ* hybridization. Either they were located outside the sections, the mRNA concentration was too low to be detected, or mRNA expression began even later in pR9.

The complimentary expression of PxUV and PxB mRNAs in pR1 and pR2 precursors in day 4 indicates that the ommatidial fate specification is complete by then (Figures 5B,E). The other photoreceptors, pR3-8, are all long-wavelength sensitive. Quite surprisingly, L opsin expression is uniform at the beginning: PxG2 mRNA is expressed in all pR3-8 cells in all ommatidia on day 5 (Figure 5K). This uniform pattern is similar to that observed in other butterfly species (Wakakuwa et al., 2004; Sison-Mangus et al., 2006; Uchiyama et al., 2013; McCulloch et al., 2016) and honeybees (Wakakuwa et al., 2005), which have only one L opsin in their genome. This simple uniform pattern persists into adulthood only in type III ommatidia in *Papilio*.

The *Papilio* pR3-8 photoreceptors are both structurally and spectrally heterogeneous. Structurally, pR3 and pR4 contribute to the distal tier of the two-tiered rhabdom, while pR5-8 form the proximal tier. The distal pR3 and pR4 photoreceptors are green sensitive in all ommatidia, suggesting they function in motion and shape vision: they form the basis for a quasi-independent achromatic system for detecting motion (Takemura and Arikawa, 2006; Stewart et al., 2015). According to Friedrich et al. (2011), the butterfly’s pR3/4 pair is homologous to fly’s dR2/5 pair, which differentiates first among dR1-6 immediately after dR8 differentiation (pR9 in the case of *Papilio*). This early differentiation may be linked to the structural and functional distinction of pR3 and pR4 in *Papilio*. The spectral heterogeneity of the pR5-8 proximal photoreceptors, which are involved in color vision (Arikawa, 2003; Koshitaka et al., 2008), is apparent by day 7. While labeling with the PxG2 probe in pR3 and pR4 remains constant, the precursors of pR5-8 lose PxG2 mRNA expression in type I and II ommatidia. These photoreceptors then express PxR mRNA strongly in type I and weakly in type II. Expression of PxG2 mRNA recovers to some extent in pR5-8 cells of type II ommatidia in the adult, which are broad-band receptors coexpressing PxG2 and PxR (Figure 1).

Because we failed to detect opsin proteins in pupal eye discs, we conclude that opsin proteins are not expressed until rhabdom formation enters its final stage on day 9: the PxG2 mRNA that is expressed transiently in pR5-8 of type I and II ommatidium on day 5 is, therefore, never translated. This correlates with the delayed rhabdom formation in *Papilio*, which happens only after the necessary opsin proteins are ready. Rhabdom formation may start earlier in the eyes of species with simpler spectral organization, which express only three basic opsins. Among lepidopterans, one such example is the silk moth *Bombyx mori* (Mita et al., 2004; Xia et al., 2004; Briscoe, 2008), where developing rhabdoms are clearly recognizable at ~50% pd. These developing eyes are even able to produce receptor potentials in response to light stimulation (Eguchi et al., 1962), which requires visual pigments.

### Perspectives from a Comparative Point of View

The observed temporal and spatial patterns of expression of opsin mRNAs in *Papilio xuthus* may have resulted from the evolution of additional opsins. Chen et al. (2016) identified opsins of ten species from four tribes in the family Papilionidae, and found that their L opsins cluster into three clades: L1, L2, and L3. These evolutionary relationships (Figure 2 of Chen et al., 2016) suggest that the ancestral L opsin was duplicated, with one opsin becoming L2 (in this case PxG2 with the peak absorption (*λ*_max_) at 515 nm) and a second opsin that later duplicated to produce L1 (PxG1 in *P. xuthus*, *λ*_max_ = 545 nm) and L3 (PxR in *P. xuthus*, *λ*_max_ = 575 nm) (Kinoshita et al., 2006). Uniform expression of the PxG2 mRNA in pR3-8 on day 5 (Figure 6) is consistent with the ancestral nature of PxG2. L1 opsins are found only in the tribe Papilionini, and at least in *P. xuthus* and *P. glaucus* L1 is always coexpressed with L2 in pR3 and pR4 photoreceptors in the ventral retina (Kitamoto et al., 1998; Briscoe, 2008). Perhaps neofunctionalization of PxG1 is not sufficient to create photoreceptors with distinct spectral sensitivity. On the other hand, the newly evolved red-absorbing PxR clearly contributes to produce red sensitive photoreceptors (Arikawa et al., 1999). The expression of PxR mRNA is always preceded by the expression of PxG2 mRNA; perhaps this order of expression simply reflects ancestral regulation, or perhaps PxR expression is itself regulated by earlier PxG2 expression.

Mechanisms that might control the temporal and spatial expression of mRNAs and the observed post-transcriptional delay in protein production remains to be studied in detail. The opsin expression pattern and the photoreceptor spectral sensitivities observed in *Papilio* (Figure 1D) are not conserved even among butterfly species. Because of this variability, comparative studies among butterflies may be a useful approach toward understanding the general principles that underlie the spatial and temporal control of opsin expression.

In the family Pieridae, M opsin duplication is common (Ogawa et al., 2012). M opsins are restricted to the pR1 and pR2, as in *Papilio*. The expression pattern is however slightly more complex and variable. The cabbage white *Pieris rapae* (Pieridae, Lepidoptera) has one S (PrUV) and two M (PrV and PrB) opsins, which are expressed in three combinations, UV/B, UV/UV and V/V. A similar case is found in the red postman *Heliconius erato* (Nymphalidae, Lepidoptera), where S opsin is duplicated (Briscoe et al., 2010; McCulloch et al., 2016). The expression of the S and M opsins must be under the control of Spineless, but the involvement of a third opsin requires at least one additional mechanism. For example, an additional step is required in *Pieris* to explain how Spineless ON-ON results in V/V, rather than B/B as in *Papilio* (Figure 1D). It cannot be that V simply takes the place of B, as this does not explain the UV/B pattern in the ON-OFF case. The situation is more complex still in the eastern clouded yellow, *Colias erate*, which has one S (CeUV) and three M (CeB, CeV1 and CeV2) opsins (Ogawa et al., 2012). CeV1 is always coexpressed with V2, and a subset of V1-V2 coexpressing cells has CeB as the third opsin; CeB is not expressed anywhere else. This pattern suggests that these M opsins are in the process of neofunctionalization, possibly like PxL1 in *Papilio*.

The most complex case ever reported is the ruddy copper, *Lycaena rubidus* (Lycaenidae, Lepidoptera), which has one S (UV) and two M (B1 and B2) opsins (Sison-Mangus et al., 2006). The authors report six types of ommatidia in *Lycaena rubidus* with all possible pairwise combinations of three opsins (UV/UV, UV/B1, UV/B2, B1/B1, B1/B2, B2/B2) in pR1 and pR2, while all other species so far studied have only three types. Moreover, B2 opsin is coexpressed in pR3-8 long-wavelength receptors with the L opsin in the dorsal region of the eye, but only in females. This is peculiar because the B2 opsin and L opsin are genetically distant, and here they break the “boundary” of the short-wavelength (pR1,2) and long-wavelength (pR5-8) photoreceptors within the ommatidia. The sex-specific “transboundary” expression of an M opsin in L receptors could be of particular interest to reveal the evolution of color vision in these flower-visiting insects.

## Author Contributions

KA, TI, MW and MK jointly conceived the project and designed the experiments. TI, MW and MK performed the experiments. KA and MK supervised the project. KA wrote the article. All authors discussed the results and manuscript.

## Conflict of Interest Statement

The authors declare that the research was conducted in the absence of any commercial or financial relationships that could be construed as a potential conflict of interest.
